# Electric Field Mediated Unclogging of Angstrom‐Scale Channels

**DOI:** 10.1002/smtd.202400961

**Published:** 2024-10-17

**Authors:** Solleti Goutham, Raj Kumar Gogoi, Hiran Jyothilal, Gwang‐Hyeon Nam, Abdulghani Ismail, Siddhi Vinayak Pandey, Ashok Keerthi, Boya Radha

**Affiliations:** ^1^ Department of Physics and Astronomy School of Natural Sciences The University of Manchester Manchester M13 9PL UK; ^2^ National Graphene Institute The University of Manchester Manchester M13 9PL UK; ^3^ Department of Chemistry School of Natural Sciences The University of Manchester Manchester M13 9PL UK

**Keywords:** 2D capillaries, 2D materials, angstrofluidics, ionic conductivity, molecular transport, nanofluidics, van der Waals assembly

## Abstract

Angstrom‐scale fluidic channels offer immense potential for applications in areas such as desalination, molecular sieving, biomolecular sequencing, and dialysis. Inspired by biological ion channels, nano‐ and angstrom (Å)‐scale channels are fabricated that mimic these molecular or atomic‐scale dimensions. At the Å‐scale, these channels exhibit unique phenomena, including selective ion transport, osmotic energy generation, fast water and gas flows, and neuromorphic ion memory. However, practical utilization of Å‐scale channels is often hindered by contamination, which can clog these nanochannels. In this context, a promising technique is introduced here for unclogging 2D channels, particularly those with sub‐nanometre dimensions (≈6.8 Å). The voltage‐cycling method emerges as an efficient and reliable solution for this challenge. The electric field effectively dislodges contaminants from the clogged Å‐scale channels, facilitating ion and molecular transport. This study provides practical guidelines for reviving clogged nano‐ and Å‐scale channels, thereby enhancing their applicability in various ion and molecular transport applications.

## Graphical abstract

Angstrom‐scale fluidic channels, with wide‐ranging applications in size‐selective separation, often face performance setbacks due to channel closure by contamination. This study introduces a voltage‐cycling technique wherein electric fields efficiently unclog these channels, thereby facilitating ion and molecular transport and extending their practical utility.

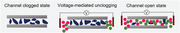

## 1 Introduction

Over the past two decades, there have been several reports on artificial nanochannels, nanopores, nanotubes, and ultra‐thin membranes with their applications in sensing, desalination, molecular separation, energy conversion, DNA sequencing, gas purification, dialysis.^[^ ^1^, ^2^, ^3^, ^4^, ^5^ ^]^ In biological systems, protein channels with Å‐scale constrictions precisely control water and ion transport through cell membranes. To achieve such precision down to the Å‐scale dimensions in the artificial channels is challenging. Sophisticated tools and the advent of new materials have helped push the boundaries in the field of nanofluidics and allowed us to fabricate systems with sub‐nanometre control on channels’ dimensions. There are many artificial ion channels with few nanometers to sub‐nanometer dimensions such as molybdenum disulfide (MoS_2_) nanopores,^[^ ^6^ ^]^ Å‐scale 2D capillaries,^[^ ^7^ ^]^ graphene nanopores,^[^ ^8^ ^]^ boron nitride nanotube,^[^ ^9^ ^]^ metal–organic frameworks,^[^ ^10^ ^]^ carbon nanotubes^[^ ^11^ ^]^ and 2D laminate membranes,^[^ ^12^ ^]^ etc. In particular, Å‐scale channels have shown intriguing phenomena such as fast water flows, ion selectivity, and ionic memory.^[^ ^2^, ^3^, ^7^, ^13^ ^]^ Such Å‐scale channels can be highly prone to contamination,^[^ ^4^ ^]^ where clogging could occur due to the adsorption of hydrocarbons present in the ambient conditions or polymeric residues from nanofabrication processes.^[^ ^4^, ^14^ ^]^ Previously, using helium (He) gas measurements, we have shown that the storage of devices in charcoal and subsequent thermal treatment can prolong their usability with open channels.^[^ ^4^ ^]^ This method of charcoal immersion has limitations in that it may not be suitable when the device needs to be measured in solutions like aqueous electrolytes where charcoal particles loosely bound on the surfaces could float in liquids and obstruct the channel entries.

It is well known that the atomically smooth basal planes of 2D materials are highly prone to contamination.^[^ ^15^ ^]^ For instance, freshly cleaved crystals of graphite, hexagonal boron nitride (hBN), and MoS_2_, showed water contact angles ranging from ≈60° to 75° but the contact angle rapidly increased (∼90°) under a few minutes of exposure to the ambient atmosphere because of adsorption of airborne hydrocarbons.^[^ ^16^ ^]^ Removing these trapped or adsorbed contaminants by heat treatment^[^ ^4^ ^]^ can open the nanofluidic channels. However, we have observed that a strong confinement below 1 nm could lead to hydrocarbon entrapment and hard to remove these hydrocarbons from the channels (especially in the case with channels height, *h* ≈6.8 Å) even after repeated heat treatments.^[^ ^4^ ^]^ The contaminants inside the nanochannels/pores obstruct the flow of ions/water, impeding ionic conductance.

In this study, we discuss methods for unclogging 2D Å‐scale channels with a confinement *h* of ≈6.8 Å. We systematically monitor the increase in conductance to track the progress of channel opening and detail our observations for various methods, including voltage cycling using electrolytes of salt solutions, acid, and water. We conduct a statistical evaluation of the voltage cycling method's effectiveness in opening Å‐scale channels from a blocked or clogged state across several devices.

## 2 Results and Discussion

**Figure 1:**
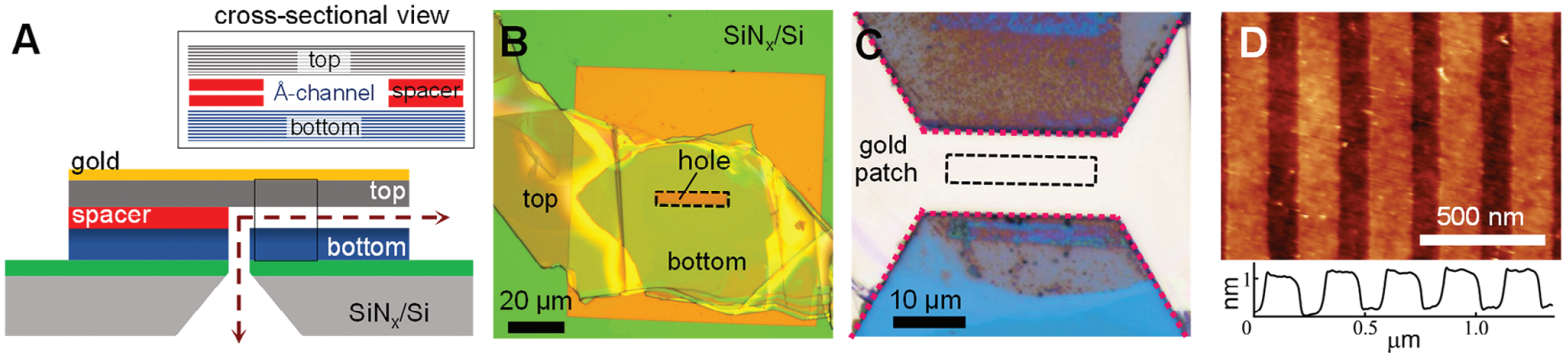
Å‐scale 2D channel devices. A) Schematic depiction of the Å‐scale 2D Channel device. The top inset shows the cross‐sectional view of the Å‐scale channel B) Optical microscopic image of Å‐scale 2D channel device. C) A gold patch was deposited onto the tri‐crystal stack, followed by a dry etching process to eliminate any uneven edges of the top crystal, and define channel length. D) An AFM topography of bilayer graphene spacers, along with a height profile on the spacers.

The Å‐scale 2D channels were fabricated using 2D materials, such as graphene, hBN, MoS_2,_ etc., as van der Waals (vdw) assemblies by following reported methods.^[^ ^2^, ^3^, ^17^ ^]^ These 2D channel devices have versatile applications in studying for ion selectivity,^[^ ^3^, ^13^, ^18^ ^]^ proton transport,^[^ ^7^ ^]^ anomalous water flows,^[^ ^2^, ^16^ ^]^ ballistic gas transport,^[^ ^4^, ^14^ ^]^ and DNA translocation.^[^ ^5c^ ^]^ Briefly, our 2D channel devices mainly consist of a tri‐crystal stack: top and bottom 2D crystals which are separated by a spacer crystal (**Figure** **1**; Section S1, Supporting Information). The top and bottom 2D crystals can be either thick (in the range of ≈100 to 200 nm) graphite or hBN or MoS_2_ crystals which are mechanically exfoliated from bulk crystals using the “scotch tape” method. Spacer crystal consists of strips of bilayer graphene sheet (i.e., *h* ≈6.8 Å) that act as spacers. Each spacer width is ≈120 nm and the distances between adjacent strips (i.e., channels’ width, *w*) range from ≈80 to ≈140 nm which acts as a channel for ion or mass transport. Each device has a specific number of channels (*n*) ranging from 1 to 400 with a channel length (*L*) of a few micrometers. The tri‐crystal stack of 2D channels was assembled on top of a several micrometer rectangular hole on silicon nitride (SiN_x_) membranes, which serves as an out‐of‐plane entry/exit of the channels as well as mechanical support as depicted in Figure 1 and Figure S1 (Supporting Information). The details of the slit‐shaped nanochannels devices used in this work are listed in Table S1 (Supporting Information). The entire nanofabrication procedure was carried out in a cleanroom (class 100/ISO 5) and after each transfer step, stacks of 2D crystals were annealed at ≈350 to 400 °C for 3 h to avoid possible contamination from process resists and polymers. Despite taking extensive measures to avoid contamination, some devices (in our case ≥ 60%) will still have trapped residual contaminates inside 2D channels, leading to clogged devices.^[^ ^17^ ^]^ The fabricated devices are thus categorized as: 1) fully functional devices that conduct gas and ions as expected and 2) clogged devices that exhibit poor ionic conductivity and gas flow due to blockage. Unclogging the contaminated channels (category 2) is an imperative need to improve the number of working devices.

**Figure 2:**
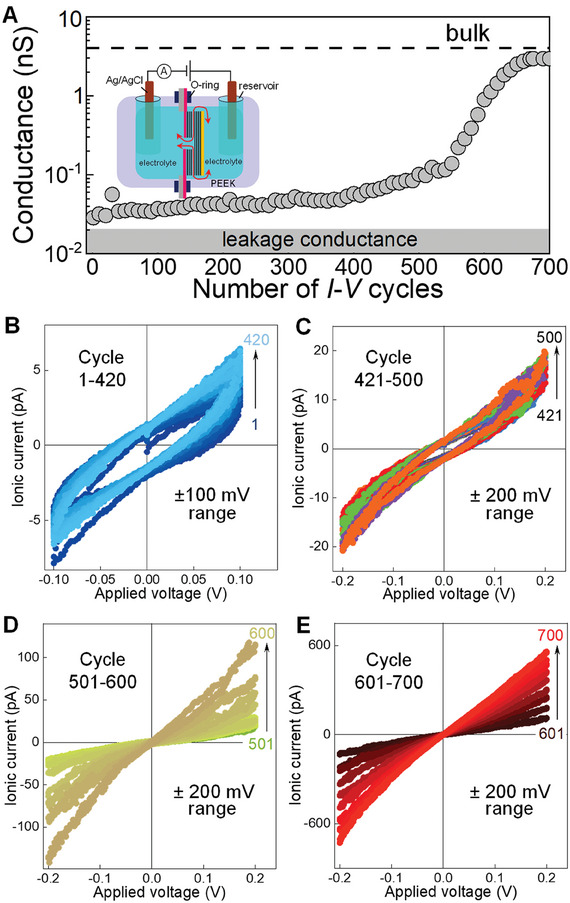
Opening of Å‐scale channels: A) The conductance *G* as a function of the number of *I–V* cycles (shown in B, C, D, and E). The grey shaded area indicates leakage *G*, and the dashed black line is the estimated bulk *G*. Every 10th *I‐V* cycle and its corresponding *G* value are presented in all the sub‐panels of this figure. Inset: Schematic of electrochemical measurement setup for ion transport measurements. B) *I–V* curves of a clogged channel device (from 1 to 420 cycles) show poor conduction at ±100 mV ranges with no significant change in the *G* with cycles. C) *I–V* curves at ±200 mV range (cycles from 421 to 500) showing less hysteresis and *G* increased in comparison to (B). D) The ionic current through the channels has improved after ≈500 *I–V* cycles (at ±200 mV) with capacitive loops reducing. E) The 2D channels are nearly open to ions after several hundred *I–V* cycles (at ±200 mV) with a steady increase in current value and finally device shows stable *I–V* characteristics after 700 cycles. The full set of *I–V* cycles are presented in Figure S4 (Supporting Information). All measurements included in this figure are conducted with 0.1 m KCl electrolyte solution using a device with top and bottom hBN walls and bilayer graphene as spacers. The dimensions of this device#9 are height, *h* = 6.8 Å, width, *w* = 110 nm, length, *L* = 5 µm and number of channels, *n*  = 190.

To confirm whether the Å‐scale channels are open or clogged, we flow He gas via custom custom‐designed measurement station (Figure S2A, Supporting Information) on the mass spectrometer. The He gas flow rate (*Q*) through these devices was compared with their theoretical value (*Q_K_*), calculated using the *Knudsen* equation (Equation S1, Supporting Information).^[^ ^14^, ^19^ ^]^ Devices exhibiting *Q* << *Q_K_* are categorized as clogged devices (Figure S2B, Supporting Information). To revive such clogged channels, devices are annealed at 350 or 400 °C for several hours (3 to 10 h) and repeated He gas flow measurements.^[^ ^4^, ^14^ ^]^ Even after this annealing, most clogged devices remain unopened. These Å‐scale channel devices (both open and clogged ones) are further tested in our electrochemical setup to check the ionic currents. The ion transport measurements are performed by using a customized electrochemical setup machined from polyether ether ketone (PEEK). This setup (**Figure** **2**A inset) allows us to measure voltage‐driven ionic currents by using Ag/AgCl electrodes and an electrical source meter (*Keithley* 2636B). First, we start with a blank device to verify if any leakage in the measurement system. This blank device consists of top and bottom 2D crystals with ≈100 to 200 nm thickness on a SiN_x_ hole without spacer crystal. The measured ionic current (*I*) upon applied voltage (*V*) ±1 V in the control experiment with a blank device in 1 m of KCl solution (see Figure S3, Supporting Information) is a few tens of pA which is translated into the leakage conductance (*G*_leakage_). In similar conditions, 2D channel devices are measured where channels are properly wet with deionized (DI) water followed by electrolyte solution, ensuring no air bubbles are present at the entry/exit of channels by slowly rinsing the solution. The bulk conductance (*G*_bulk_) is calculated from the bulk conductivity (*σ*_bulk_)^[^ ^20^ ^]^ of the particular electrolyte solution as *G*_bulk_ = *σ*_bulk_ (*nhw*/*L*), where *n*, *h*, *w*, and *L* are the number of channels, height, width and the length of the channels, respectively.^[^ ^3b^ ^]^ The devices with measured *G* << *G*_theory_ by less than an at least order of magnitude are categorized as clogged devices.

### 2.1 Unclogging of Å‐Scale Channel by *I–V* Cycling

To open clogged Å‐scale channels, we do *I–V* cycling by mounting a clogged device into the electrochemical setup (shown in the inset of Figure 2A) and filling the reservoirs with the desired electrolyte solutions. This device has hBN crystals as top and bottom walls with *n* = 190, *h* = 6.8 Å, *w* = 110 nm and *L* = 5 µm. First, water was used as an electrolyte to record the *I–V* curves through the Å‐scale channels, which led to low *G* (few pS close to the *G*_leakage_, shown as leakage conductance in Figure 1A). Next, the *I–V* characteristics were measured for 0.1 m KCl solution, using voltages of ±100 mV (voltage step, 2 mVs^−1^) with several repeating loops. The variation of *G* with the increasing *I*–*V* cycles is shown in Figure 2A. The initial *I*–*V* curve (in Figure 2B) measured with 0.1 m KCl has a large capacitive loop and low ionic currents, indicating that the channels are not yet open (negligible ion conduction). A hundred repeated *I–V* cycles only led to a partial increment of *G*, Figure 2A,B. After the initial hundred cycles, the measured *G* remained close to the *G*_leakage_ (see Figure 2A). At this stage, the measurements are stopped, and the salt solutions are removed from the reservoirs. The reservoirs are then sequentially rinsed with DI water, a 10% isopropyl alcohol (IPA) solution in water, and finally with pure DI water. Fresh 0.1 m KCl solution is then refilled into the reservoirs to perform the next 320 *I–V* cycles within a voltage range of ±100 mV. As there is no significant difference found in the *I–V* loops (Figure 2B), then the voltage range is increased to ±200 mV and the *I–V* cycles are continued for another hundred loops (Figure 2C) with fresh 0.1 m KCl solution. Until the channels are open and display significant differences in *I–V* loops, the same procedure (rinsing with DI water and IPA) is repeated for about every hundred *I–V* cycles. At each repetitive washing step, the IPA solution concentration is increased by 10%. After several (4 to 5) repetitive rinsing‐measuring steps, *G* started to increase as shown in Figure 2D,E. The *I–V* curve still has hysteresis but a steady *G* which increased within consecutive cycles Figure 2A. Subsequently, after ≈600 cycles, the *G* raises (Figure 2A) continuously, and eventually within a few cycles, it becomes stable. Further continuation of *I–V* cycles, the channels’ *G* has reached saturation, which is approximately close to the calculated *G*_bulk_ value.^[^ ^3b^ ^]^ The stable current (or *G*) proves that most of the channels were opened. The voltage cycling is to be done at least in the ±200 mV range as lower voltage cycling at ±100 mV range for 1000 cycles (Figure S8, Supporting Information) is usually not effective in opening channels. Once the devices are unclogged by an electric field, the channels allow water, gases as well as ionic solutions. At this stage, we conducted helium gas flow measurements once again to determine the flow rate through the channels (Figure S2B, Supporting Information). The increased He flow rate is compared to the starting value before the voltage cycling, also confirms that the channels are open.

The contaminants trapped inside the channels act as barriers to the ion pathways, hindering the ionic flow. The voltage cycling, which varies the electric field across the channel, facilitates the repeated accumulation of ions at the channel entrances. The electric field helps in creating percolation paths through the contaminants, eventually allowing the ions to penetrate them. With multiple voltage cycles, the ions progressively clear the path by pushing out the contaminants, allowing the uninterrupted flow of ions.

As our channels can have different wall materials, we repeated this *I–V* cycling method for channels with graphite and MoS_2_ as the channel walls. Although the channels are made from different materials, they exhibit relatively low surface charge densities of a few hundred µC cm^−2^ due to the pristine nature of the mechanically exfoliated flakes used to construct them. In addition, to avoid the effects of surface charges, we used an ionic concentration of 0.1 m, which is above the surface charge‐governed regime for these channels. Clogged devices with all these materials as channel walls could be opened with no significant differences (shown in Figure S5, Supporting Information).

**Figure 3:**
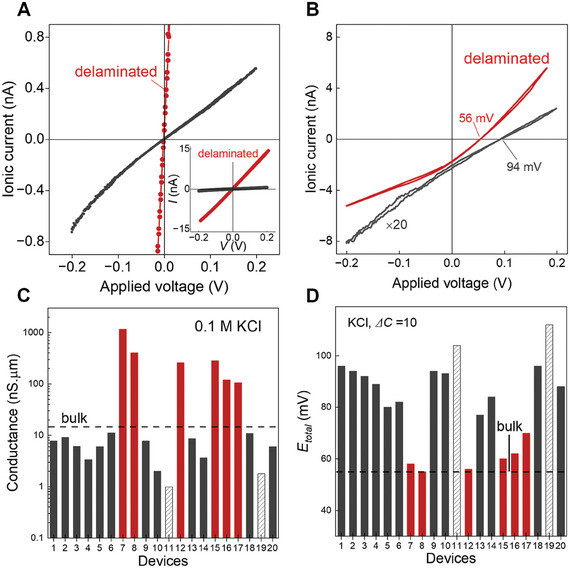
Conductance and membrane potential measurements for several Å‐channel devices. A) *I–V* measurements when the channels are intact and in a lifted/delaminated state. The inset shows the full current range of the delaminated channels. B) Drift‐diffusion experiments when the device is intact and delaminated state under a concentration gradient *ΔC* = 10 for KCl solution. For comparison, the diffusion *I*–*V* curve of the intact channels is magnified by 20 times. C) Measured conductance *G* of twenty different devices (similar *h* ≈ 6.8 Å, *w* ≈ 120 nm) in 0.1 m KCl solution. For comparison amongst several devices, the G values are normalised to *n* = 100, and *L* = 1 µm. D) The obtained *E*_total_ from drift‐diffusion experiments with *ΔC* = 10 for KCl solution on the same devices as in panel C. In C and D, red bars are delaminated devices, the black dashed line indicates bulk values for *G* and redox potential (i.e., 55 mV). The same device in Figure 2 is used here in A and B and denoted as device #9 in C and D. Devices #11 and #19 have relatively high membrane potential, which is likely due to the channels not being fully open.

One of the important characteristics of these working channels with *h* = 6.8 Å is selective ion transport. To compare the ion selectivity of the unclogged devices with stable *G* (open state after 700 cycles, Figure 2E), we perform drift‐diffusion experiments (**Figure** **3**) under a concentration gradient Δ*C* = 10 for KCl, as a cross‐check^[^ ^3^, ^13^ ^]^ (see Section S3, Supporting Information). For this device, the obtained zero current potential (*E_total_*) (which includes redox potential, *E*_redox_ of ≈55 mV) is found to be ≈94 mV which indicates cation selectivity.^[^ ^3^, ^13^ ^]^ The ion selectivity in our channels arises due to the confinement being close to the size of the ions.^[^ ^3^, ^13^ ^]^ Based on the *G* and diffusion experiments, we confirm that the device is in working condition with open channels. If the device were to lift off or delaminate, i.e., the presence of non‐confined or artifact pathways for the electrolyte to permeate between the three layers or through the stack‐hole interface rather than the Å‐scale channels (see Supporting Information for further details), the device would display a high current (Figure 3A and inset). Additionally, in the case of delaminated channels, the drift‐diffusion (Figure 3B) leads to a decreased membrane potential from ≈94 to 56 mV, which matches the bulk *E*_redox_ calculated from Equation (1).^[^ ^21^ ^]^

(1) $$E_{\mathrm{redox}}=\frac{RT}{zF}\;In\left(\frac{\gamma_{H}C_{H}}{\gamma_{L}C_{L}}\right)$$

where *R* is the universal gas constant, *T* is the temperature, *F* is Faraday's constant, *z* is the charge number, γ_H/L_ and *C*_H/L_ are the activity coefficient and the concentration of salt solution, respectively, on the high (*H*) and low (*L*) concentration sides. A correlation of the Å‐channel opening on its drift–diffusion (zero–current potential) is shown in Figure S6 (Supporting Information).

To check the reproducibility of our channel opening method, we have studied several devices and compared the *G* (see Figure 3C) after normalizing the *G* with 1 µm channel length for 100 channels to enable comparison amongst devices. The majority of the open devices show *G* slightly lower or close to that expected for bilayer channels,^[^ ^3^, ^13^ ^]^ while some of them are delaminated with high *G* (red color bars, Figure 3C) and others had partially opened channels. To double‐check the device status, we have performed drift–diffusion experiments on all of them, and the corresponding membrane potential (*E*_total_) values are shown in Figure 3D. With the channel confinement height of 6.8 Å close to the ions size, we have previously reported the ion selectivity between K^+^ and Cl^−^ ions which is reflected in the increase of the membrane potential relative to bulk redox, as Cl^−^ ion mobility was suppressed. In the devices with higher *G*, the membrane potential is close to the redox value (Figure 3D), which means the height of the channel is no longer confined and hence no selectivity of ions. For instance, device #7 *G* is very high and its membrane potential is close to the bulk, indicating that the device was delaminated (Figure 3C,D). In few of the devices where channels are not fully open (e.g., device #4), although *G* was lower than expected, the corresponding membrane potential is indicative of selective channels, meaning such devices had few open channels. Devices #11 and #19 have higher *E*_total_ values in comparison to the other functional devices. This might be due to the capacitive nature of the *I–V* curves (Figure S7, Supporting Information) leading to two values of *E*_total_ at *I* = 0; the mean of these values is represented in Figure 3D. Thus, membrane potential can be an additional cross‐check to know if the Å‐channels are open and functional or delaminated.

We have also used our voltage cycling method to open single‐channel devices and demonstrated its versatility in unclogging angstrom‐scale channels (Figure S9, Supporting Information). Discrete increments in the conductivity are often observed in both multi‐channel and single‐channel devices during the unclogging process. This can be attributed to the sudden removal of contaminants from the channels. Such single‐channel devices are useful for translocation, sensing, and sequencing of biomolecules such as DNA and protein.^[^ ^5c^ ^]^ Similar to a multi‐channel device, a single‐channel device is unclogged successfully with *G* values closing matching to *G*_bulk_ after ≈750 *I–V* cycles (400 cycles of ±100 mV and 350 cycles of ±200 mV *I–V* cycles, see Figure S9, Supporting Information).

### 2.2 Effect of Applied Potential on the Unclogging of Å‐Scale Channel

**Figure 4:**
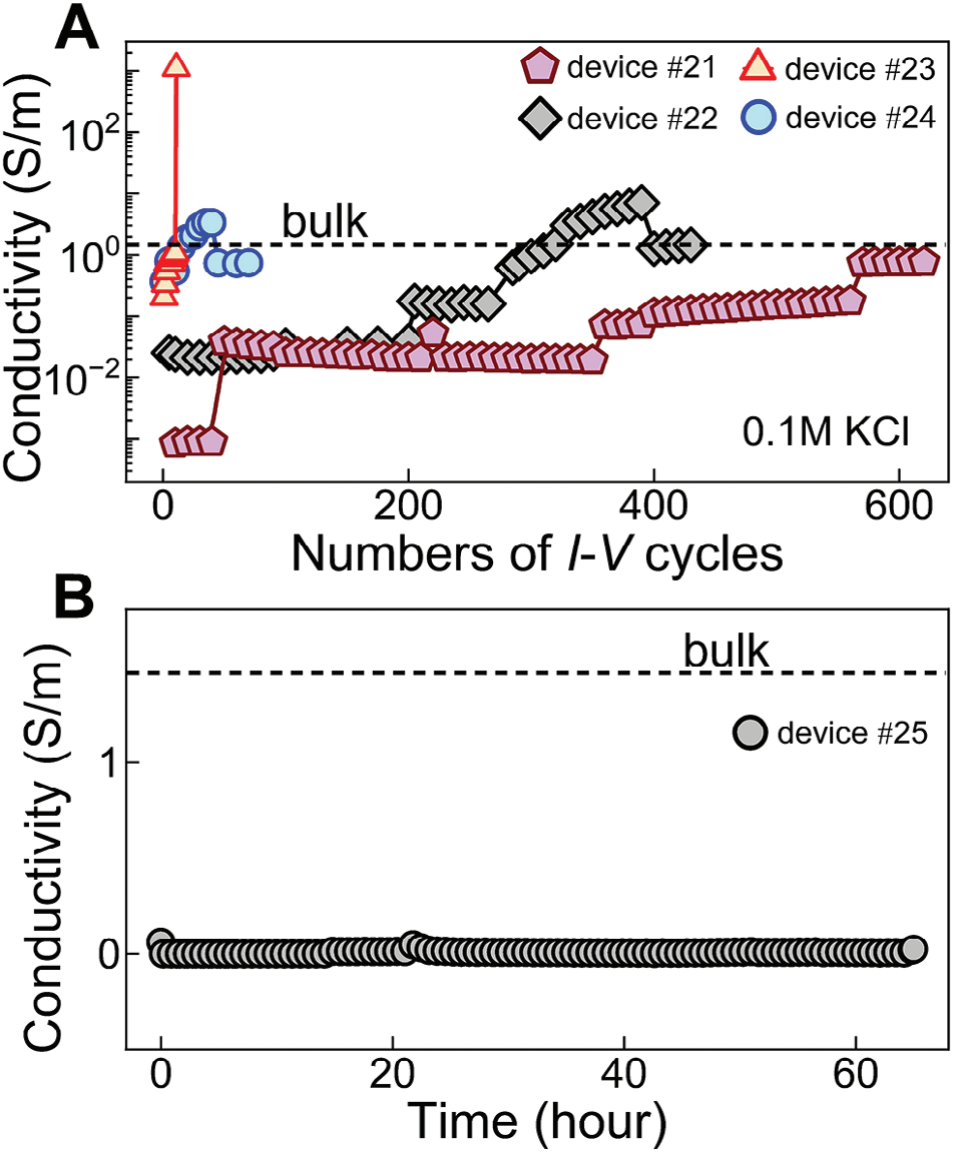
Voltage variation for opening Å‐channel devices. A) High voltage (±500 mV to ±1 V) cycling for opening the channels monitored by increasing conductivity with a number of *I–V* cycles (0.1 m KCl solution). Device #21 was subjected to ±200 mV for 50 cycles followed by ±500 mV until ≈550 cycles, followed by ±1 V in the subsequent cycles. In device #22, the initial 200 cycles were measured at ±200 mV and the following cycles were measured at ±800 mV. For devices #23 and #24, we start measuring at ±1 V. Black color dashed line indicates bulk conductivity of 0.1 m KCl. B) Conductivity as a function of time for a 0.1 m KCl solution, measured at a constant voltage of 100 mV for 22 h (equivalent to the time required for completing 400 *I–V* cycles within a ±100 mV range). After 22 h, a voltage of 200 mV was applied for another 44 h (equivalent to the time required for completing 400 *I–V* cycles within a ±200 mV range). The black color dashed line indicates the expected conductivity value.

We have investigated the effect of the applied voltage on the unclogging process by using different voltage ranges during the *I–V* cycling process. To perform this experiment, four clogged Å‐scale channel devices were chosen and measured the resulting *I–V* cycles with 0.1 m KCl as electrolyte (devices #21, #22, #23, and #24, **Figure** **4**A). For comparison, we calculate conductivity from the measured *G* values by using $\sigma =\frac{GL}{hwn}$. With device #21 (Figure 4A), we applied ±200 mV for the first 50 cycles, where σ was significantly less than *σ*_bulk_, indicating clogged channels. Increasing the applied voltage to ±500 mV raised σ by an order of magnitude compared to that at ±200 mV. However, even after ≈350 cycles, *σ* remained 100 times lower than *σ*_bulk_. Further cycling at ±500 mV led to an increase in *σ*, reaching a value one order lower than *σ*_bulk_ after ≈550 cycles. Increasing the voltage range to ±1 V eventually resulted in σ values comparable to *σ*_bulk_. To test the stability of the devices under high voltage, device #22 (Figure 4A) was subjected to ±800 mV after the initial 200 cycles at ±200 mV. The *σ* began to increase after 80 *I–V* cycles at ±800 mV, attaining *σ* = *σ*_bulk_ after ≈550 *I–V* cycles. This *σ* = *σ*_bulk_ was retained when the device was subsequently cycled at ±200 mV. Since high voltage can unclog devices more quickly, we tested cycling devices at ±1 V. We limited the voltage range to 1 V or below to prevent any delamination or damage to the channel devices. For device #23, after ≈10 *I–V* cycles, the conductivity abruptly increased to a very high level (*σ*/*σ*_bulk_ = 1000, Figure 4A) and did not recover at lower voltage ranges, indicating delamination tri‐crystal stack or top crystal from the stack on SiN_x_ substrate.^[^ ^20^ ^]^ To verify this, we repeated the experiment with device #24. The measured conductivity, *σ* started to increase after a few cycles and reached *σ* = *σ*_bulk_ after ≈15 *I–V* cycles. Similar to device #22, further high‐range cycling resulted in *σ* > *σ*_bulk_, and cycling back at lower ranges returned *σ* to *σ*_bulk_.

Next, we explored channel opening using a constant voltage instead of cycling. We used another clogged device, #25, and 0.1 m KCl solution as electrolyte. A constant voltage of 100 mV was applied continuously while measuring ionic current, which is translated into conductivity in Figure 4B. This experiment was performed for over 22 h, and no substantial change in σ was observed; it remained close to zero. Every 5 h, we washed the channel devices with water and IPA and replaced the solution with fresh 0.1 m KCl solution to ensure consistency in the studied parameters. Later, we increased the applied voltage to 200 mV and measured ion conductivity over 44 h (Figure 4B). Over a total of 66 h of measurements, no significant change in conductivity was observed, and the channels remained in a closed state. This duration is equivalent to 800 *I–V* cycles (400 cycles of ±100 mV and 400 cycles of ±200 mV). Unlike the case of constant voltage application, *I–V* cycling leads to a variable electric field, where ions repeatedly try to enter the channels and contact the contaminants, gradually creating defect paths and eventually entering the channels. Over multiple *I–V* cycles, the ions push the contaminants out, reducing resistance in their transport paths and ultimately leading to the opening of the channels. Thus, voltage cycling proved to be more efficient than constant voltage application for opening the channels.

### 2.3 Effect of Electrolyte Solution

**Figure 5:**
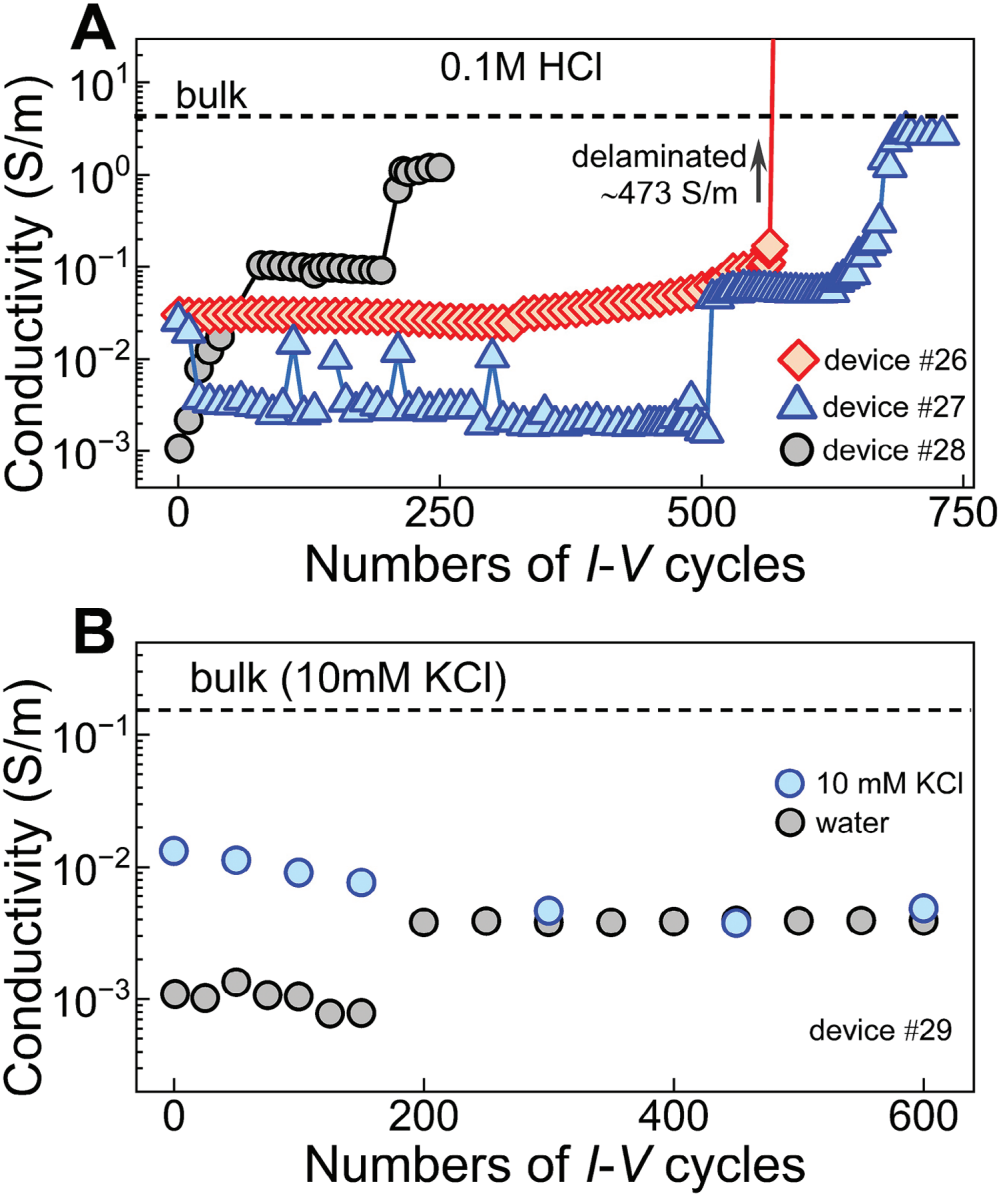
*I–V* cycles of Å‐channel devices with HCl and water. Conductivity versus the number of *I–V* cycles for A) 0.1 m HCl solution and B) water. In (A) with HCl, device #26 delaminated after 500 *I–V* cycles, and device #27 successfully opened at ≈700 *I–V* cycles, for both devices *I–V* cycling was performed at ±200 mV. For device #28, the first 120 cycles were performed at ±200 mV followed by the subsequent cycles at ±1 V. For every 100th cycle devices undergo rinsing with IPA/water. In (B) with water, there was no increase in conductivity after 600 *I–V* cycles. To verify channel opening, we conducted *I–V* measurements of 10 mm KCl after every 50 *I–V* cycles. The dashed black lines in (A) and (B) indicate bulk conductivities of 0.1 m HCl and 10 mm KCl respectively.

We further examined the influence of other electrolytes and the concentration of electrolytes on the unclogging of Å‐channel devices. We specifically chose 0.1 m HCl as an electrolyte as protons can diffuse faster than K^+^ ions, and tested this with three devices (**Figure** **5**A). Device #27 when cycled in the ±200 mV range with 0.1 m HCl, unclogged after ≈600 cycles, attaining *σ* = *σ*_bulk_. However, with the same experimental conditions, device #26 exhibited an abrupt jump in the *σ* to ≈473 S m^−1^ just after 500 cycles indicating the delamination of the device. As HCl can be used to unclog the fluidic channels, a clogged device #28 has been subjected to ±1 V range *I–V* cycles. Initially, device #28 (Figure 5A) was subjected to ≈120 cycles (±200 mV, range), and the measured *σ* << *σ*_bulk_, indicating it was in a blocked stage. When the device was further cycled at ±1 V, it was unclogged after ≈200 cycles (≈120 cycles of ±200 mV and ≈80 cycles ±1 V). Furthermore, we avoid using lower electrolyte concentrations (< 0.1 m) as low ion flux would require longer voltage cycling time to effectively push contaminants out of the channels for unclogging.

Next, we tested water as an electrolyte to open the clogged Å‐channel. For this, we used another clogged device (#29) and filled DI water into reservoirs to do *I–V*. Even for open Å‐channels, water σ is much smaller compared to that of salts. We initially measured the conductivity of 10 mm KCl. It is evident that the channels are completely blocked and exhibit poor conduction. The choice of 10 mm KCl for monitoring the *σ* instead of 0.1 m KCl is aimed at minimizing the influence of salt high concentration on device conductance. We performed 600 *I–V* cycles with water, and at every 50‐cycle interval, we cross‐checked the conductivity with 10 mm KCl. There were no significant changes in the channels’ conductivity (Figure 5B), thus indicating that the device remains in a clogged state and that water is not suitable as an electrolyte to open the channels.

The experimental findings in this work highlight the efficacy of the *I–V* cycling method in unclogging and opening the Å‐scale 2D channels. By voltage cycling, the ions are subjected to an electric field driving their movement from one end of the channel to another. The contamination trapped inside the confined channels blocks ions prohibiting their transport. Upon applying an electric potential, the ion flow pushes the contaminates over time and eventually unclogs the channels. All the methods described in this work can be employed to unclog the devices, but the opening at a lower voltage range (i.e., ±200 mV) is preferred over the higher ones. At high voltages (electric fields), a greater number of ions tend to enter the channel, accelerating the unclogging process. However, the contaminants can also act as a barrier against the ion flows, leading to a sudden expansion of channels or delamination of the 2D‐crystals stack at high electric fields. Therefore, a low voltage is preferable for unclogging the channels, as it reduces the probability of delamination by allowing fewer charged species to enter. In the case of HCl and KCl aqueous electrolyte solutions, the electrolytes dissociate in water to form ions and aid in opening the channels over time under the electric field. The electrolyte solutions can impact the unclogging process. For instance, between KCl and HCl, HCl is more effective at clearing blockages due to its higher proton mobility and smaller hydration radius. At the same time, the use of HCl increases the probability of delamination due to the rapid movement of ions and the weakening of the van der Waals adhesion between the layers or of the stack from the substrate. In contrast, water, lacking ionic species, is ineffective in the unclogging process.

As a working sequence or methodology, when one starts with a clogged device, if it remains clogged after doing ±200 mV (using 0.1 m KCl) for > 700 *I–V* cycles, it can be further subjected to a high voltage (up to ± 1 V) range *I–V* cycling using 0.1 m HCl or KCl for unclogging, where majority of the devices get unclogged. Figure S10 (Supporting Information) shows a device that remained clogged until 800 cycles at ±200 mV with 0.1 m KCl. Afterward, the electrolyte was replaced with 0.1 m HCl and the voltage range was increased to ±1 V. A sudden increase in the *σ* was observed after 140 cycles of 0.1 m HCl when cycled in the voltage range of ±1 V (i.e., 940 cycles in total). The device was then washed with DI water several times and was further measured with 0.1 m KCl at the voltage ±200 mV, which showed increased *σ* but still lower compared to *σ*_bulk_, indicating the device is not delaminated even after harsh treatment. If the aforementioned methods fail to unclog the device, we proceed with one of the following approaches. First, the device is subjected to 100% IPA, followed by *I–V* measurements. Alternatively, we perform concentration gradient *I–V* measurements under varying concentration gradients. The devices which remain unclogged after subjecting to all the methods are considered defective devices.

## 3 Conclusion

In conclusion, we employed various approaches to unclog channels with a height of 6.8 Å. These approaches included the *I–V* cycling, constant voltage application, and use of different electrolytes. Both HCl and KCl solutions (0.1 m each) as electrolytes successfully opened the clogged devices through *I–V* cycling, either at ±200 mV or within high voltage ranges (±500 mV to ±1 V). However, cycling at a lower voltage range, ± 100 mV, did not unclog the devices. Cycling at ±200 mV voltages requires a longer duration for unclogging, whereas higher voltages require a shorter period. Once unclogged by these methods, the transition from a non‐conducting state to a conducting state was confirmed through ion and gas permeation. Size‐selective ion transport of Å‐channels was thus successfully restored, as verified by the drift‐diffusion experiments. The *I–V* cycling method is also successful in opening single‐channel devices. Our method of unclogging the channels can restore ≈60% to 70% of the clogged devices. This electrophoretic ion flow based unclogging/opening method could be generally applicable for various angstrom‐scale channels of different dimensionality such as pores, tubes, and porous membranes, and holds promise as a significant development for the angstrom‐scale fluidic channel devices’ applicability and for rejuvenating the clogged devices.

## Conflict of Interest

The authors declare no conflict of interest.

## Author Contributions

B.R. and S.G. designed and directed the project. S.G. led the development of the channel opening techniques. A.K., G.H.N., and H.J. carried out the sample fabrication of Å‐channel devices. S.G. and R.K.G. performed the ion conductance measurements and their analysis. A.K. and H.J. carried out the sample characterization. B.R. and S.G. wrote the manuscript with inputs from A.K., R.K.G., and A.I., and all the authors contributed to discussions.

## Supporting information



Supporting Information

## Data Availability

The data that support the findings of this study are available from the corresponding author upon reasonable request.
