# Supplementary material for: Electric Field Mediated Unclogging of Angstrom‐Scale Channels
Source: Small Methods. 2024 Oct 17;9(3):2400961. doi: 10.1002/smtd.202400961 (PMC11926513; doi:10.1002/smtd.202400961)
Supplement: Supplementary file 1 — Supporting Information [file SMTD-9-2400961-s001.pdf]

small methods

Supporting Information

for *Small Methods*, DOI 10.1002/smtd.202400961

Electric Field Mediated Unclogging of Angstrom-Scale Channels

*Solleti Goutham, Raj Kumar Gogoi, Hiran Jyothilal, Gwang-Hyeon Nam, Abdulghani Ismail, Siddhi Vinayak Pandey, Ashok Keerthi and Boya Radha**

Supporting information for

Electric field mediated unclogging of Angstrom-scale Channels

Solleti Goutham[1,2], Raj Kumar Gogoi[1,2], Hiran Jyothilal[1,2], Gwang-Hyeon Nam[1,2], Abdulghani Ismail[1,2], Siddhi Vinayak Pandey[1], Ashok Keerthi[2,3], Boya Radha[1,2*]

[1]Department of Physics and Astronomy, School of Natural Sciences, The University of Manchester, Manchester M13 9PL, United Kingdom
[2]National Graphene Institute, The University of Manchester, Manchester M13 9PL, United Kingdom
[3]Department of Chemistry, School of Natural Sciences, The University of Manchester, Manchester M13 9PL, United Kingdom

* Correspondence to be addressed to: radha.boya@manchester.ac.uk

Contents:

S1. Device fabrication

S2. Helium gas flow measurements.

S3. Ion transport measurements

Figs. S1 to S9

References

S1. Device fabrication

In the present study, we have used mainly bilayer graphene channel devices (h = ~6.8 Å) and the fabrication procedure is as in our previous reports.[1] We used commercially available 2D bulk crystals (Manchester nanomaterials Ltd.) and mechanical exfoliation was done using scotch tape on SiO_2/Si wafer with the oxide layer thickness of ~290 nm. The 2D flakes (~50 nm thick) transferred by PMMA assisted flake-transfer process onto a SiN_x membrane with a predrilled 25 µm × 3 µm hole (Fig. S1A). Dry etching was done from the back of the hole to etch bottom crystal (oxygen plasma for graphite, CHF_3/oxygen plasma for hBN). Further, a bilayer graphene flake was exfoliated onto another Si/SiO_2 substrate to be used as a spacer which defines the height (h = ~6.8 Å) of the channels. The spacer graphene flake was patterned into parallel strips (width ~120 nm ± 10 nm with few microns length) using e-beam lithography. The distance between the adjacent spaces are in the range of ~80 nm to 140 nm. The spacer strips were then transferred on to bottom crystal (Fig. S1B), and a back etch was done again to open through the spacers in the microhole. A thick (~150 – 200 nm) 2D crystal was transferred on to the spacers, which results in channels formation (Fig. S1C). At every transfer step, annealing was done at 350 °C or 400 °C for 3 hours to eliminate possible contamination.[2] Finally, Au/Cr (5/60 nm) thin film was deposited using thermal oe e-beam evaporator on the top crystal following photolithography to serve as a mask (Fig. S1D). Dry etching was done in unmasked region to define the channel lengths. In this study we have used graphene, hBN and MoS_2 as walls and bilayer graphene spacers.

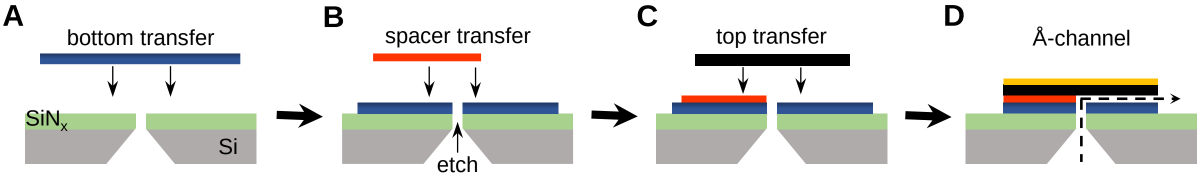

Fig. S1: Å-scale channels fabrication procedure. (A) Bottom 2D crystal was transferred on to a pre-drilled rectangular hole (25 µm × 3 µm) on SiN_x membrane, later back etch was performed to extend the rectangular hole. **(B)** Bilayer graphene strips with ~120 nm ±10 nm wide and spacing between adjacent strips of ~130 nm ±10 nm were transferred on to bottom flake on SiN_x substrate. Further, back etch was performed to remove exposed strips on the hole area. **(C)** Thick (~150-200 nm) 2D crystal transferred. **(D)** A metal film (Au/Cr) coated on the tri-crystal stack, and etching was done using it as a mask. The spacers are on either sides of the hole on the top of the SiNx membrane, however in the schematic they are shown on only one side for clarity.

S2. Helium gas flow measurements

A specially designed in-house sample holder was used to mount the Å-scale channels, which makes sure that He gas passes exclusively through Å-scale channels on our devices. This sample holder's top portion was connected to the chamber where He gas was introduced and feed chamber pressure can be monitored through a pressure gauge. The bottom chamber of the sample holder was directly connected to a mass spectrometer (He leak detector). This bottom chamber was maintained at the 10^{-3} bar pressure before every measurement. After each measurement, we evacuate both chambers with the help of a vacuum pump. A schematic illustration of the experimental set up is shown in Fig. S2A.

The theoretical mass flow (Q_K) through a 2D channel can be estimated using the Knudsen equation:[3]

$$Q_K = \alpha P \left(\frac{m}{2\pi RT}\right)^{\frac{1}{2}} wh \qquad \text{(S1)}$$

where m is the atomic mass of the gas being transported, P is the pressure, R is the gas constant, T is the temperature and w and h are the width and height of the channels. For 2D channels with $l > w$, α can be approximated [1d] as:

$$\alpha \approx \frac{h}{l} ln\left(\frac{4w}{h}\right) \qquad \text{(S2)}$$

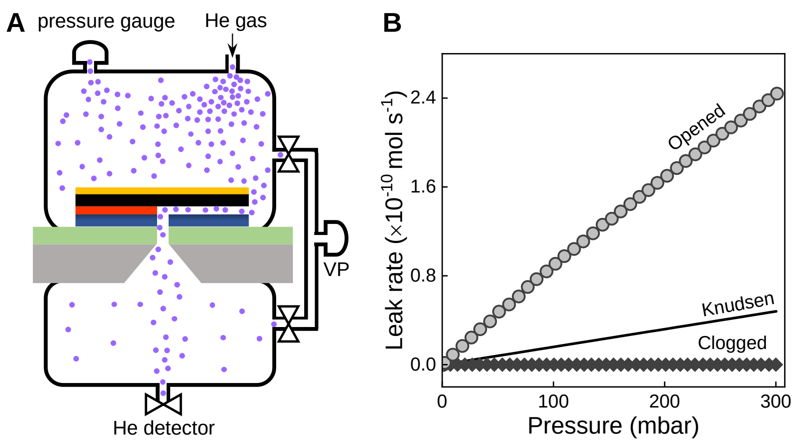

Fig. S2: He gas flow. (**A**) Schematic illustration of the in-house gas measurements setup. For the detection of He gas, commercially available leak detector was used from *Leybold, PHOENIX (Quadro dry)*. Vacuum pump is connected to both chambers of sample holder for evacuation of gas. Pressure gauge is used to monitor the pressure inside the feed (top) chamber. **(B)** Comparison of He leak rate for Å-scale channels device in the blocked and unclogged stages with the theoretical (Knudsen) flow rate. Device dimensions: $h \sim 0.68$ nm, $w \sim 110$ nm, $L \sim$ 5μm, $n \sim 190$.

S3. Ion transport measurements

To measure *I-V* characteristics, customised setup was machined from polyether ether ketone (PEEK) material; with two reservoirs to fill the electrolyte solution as well as to insert Ag/AgCl electrodes. After filling the reservoirs with 2.5 ml of electrolyte the reservoirs are sealed to prevent any solution evaporation. Freshly prepared Ag/AgCl electrodes were used for each experiment. The *I-V* characteristics were recorded using Keithley 2636B by interfacing with LabVIEW. After each measurement, deionized (DI) water was used to wash the entire cell to remove any residual salts. Each voltage cycle with ±200 mV range took about 6.6 minutes to complete including the communication time for recording the measurement.

In drift-diffusion measurements, two reservoirs filled with two different concentration solutions (concentration difference, Δ=10 in our case with 10mM || 100mM KCl) with equal volume. The working electrode is immerged in the low concentration chamber and the counter electrode is placed in the high concentration chamber. Then zero-current potential (E_{total}) was measured from the *I-V* characteristics. E_{total} is a combination of redox potential, E_{redox} (a potential arising due to redox reactions on Ag/AgCl electrodes) and membrane potential, E_m. Thus, the E_m can be estimated as:

$$E_m = E_{total} - E_{redox}$$

Delamination of A-scale channels devices:

In our channel devices, delamination refers to positional changes within the tri-crystal stack or between the stack and the micro-hole, leading to the creation of additional pathways for fluids to permeate, rather than through the Å-scale channels. This compromises the confinement effect and results in increased ionic currents that exhibit bulk-like behavior. Delamination is a common issue in freestanding 2D-materials on a membrane, primarily due to the reliance on van der Waals forces[4]. To mitigate this problem, additional supports, such as metal deposition (in our case, gold patch), have been used to clamp the 2D channels on the membranes as shown in Fig. 1C.

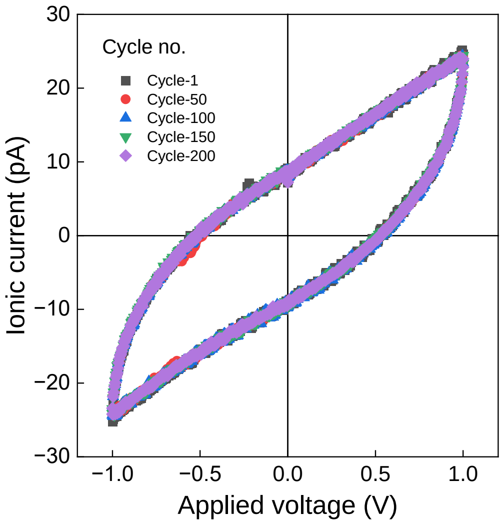

Fig. S3: *I*-*V* measurements of a blank device (top and bottom stack without a graphene spacer) using 1M KCl at an applied voltage range of ±1 V.

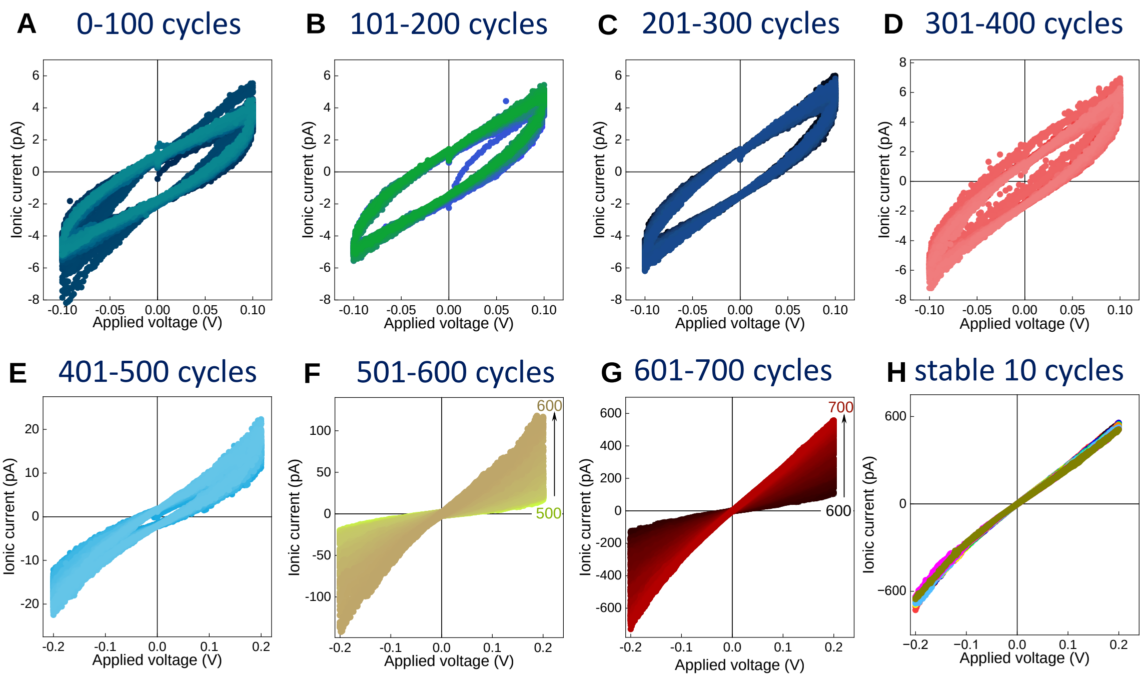

Fig. S4: Full range *I*-*V* cycles of 0.1 M KCl, transformation from non-conducting to conducting state of Å-scale channel. (A – G) 700 *I*-*V* cycles were recorded for 0.1 M KCl solution, steady increase in currents was found between ±0.1 and ±0.2 V range. A clear trend was observed from poor ionic conduction state to fully functional (opened) conduction state. **(H)** Once open, the Å-scale channel stable conductance was shown for 10 consecutive cycles. The dimensions of the device #9 are height, $h \sim 6.8$ Å, width, $w \sim 110$ nm, length, $L \sim 5$ μm and number of channels, $n \sim 190$.

Table S1: Details of the devices utilized in this study. Channel height, h ~6.8 Å (2-layer graphene spacer) remains same for all the devices.

Device number	No. of channels, N	Channel length (μm)	Channel width (nm)	Wall material
#1	150	8	130	Graphite
#2	150	7	130	hBN
#3	100	8	130	hBN
#4	200	7	130	Graphite
#5	88	7	130	MoS_2
#6	200	2.5	130	Graphite
#7	300	2	130	Graphite
#8	400	1	130	Graphite
#9	190	5	110	hBN
#10	200	2.6	130	Graphite
#11	300	6.5	130	MoS_2
#12	160	5	130	Graphite
#13	208	5.3	130	hBN
#14	170	5.2	130	hBN
#15	80	4.5	130	hBN
#16	230	6.8	130	hBN
#17	200	3	130	hBN
#18	180	6.1	130	hBN
#19	200	9	130	Graphite
#20	210	3	130	hBN
#21	188	4.7	130	hBN
#22	198	6.5	130	MoS_2
#23	190	5.1	130	hBN
#24	180	8	130	Graphite
#25	200	5	110	hBN
#26	176	5.6	130	MoS_2
#27	200	5	130	MoS_2
#28	236	7	130	MoS_2
#29	390	5	130	hBN
#30	250	5.5	130	Graphite
#31	320	6.5	130	MoS_2
#32	380	4.8	100	hBN
#33	198	4.5	110	Graphite

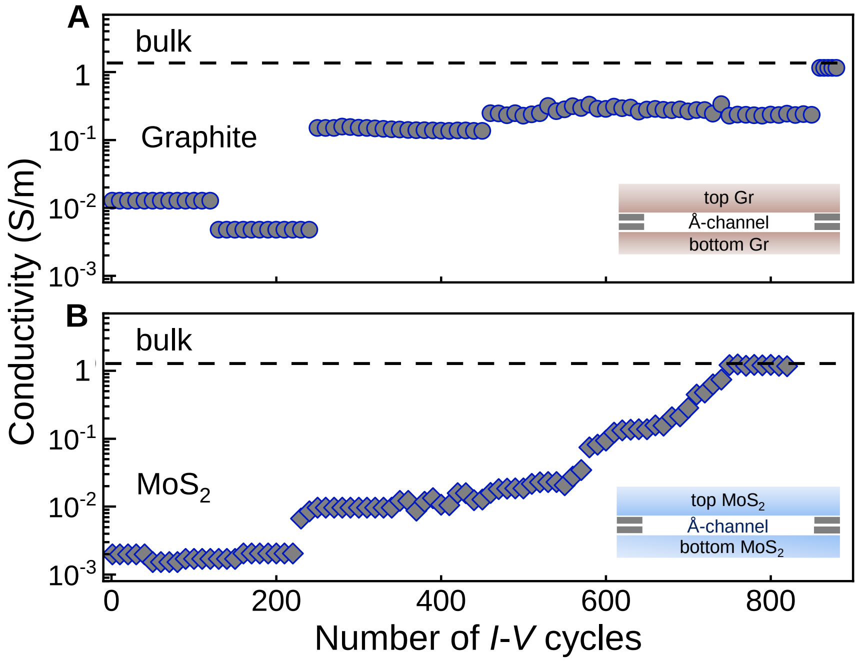

Fig.S5: Influence of wall materials on unclogging of Å-scale channels. Conductivity *vs* number of *I-V* cycles (0.1 M KCl) for two different devices containing channels of height $h \sim 6.8$ Å, with top and bottom walls of **(A)** graphite ($n \sim 250$ channels in parallel; $w \sim 130$ nm and $L \sim 5.5$ µm, device #30) and **(B)** MoS_2 ($n \sim 320$ channels in parallel; $w \sim 130$ nm and $L \sim 6.5$ µm, device #31). Dashed black lines are the bulk[5] 0.1 M KCl conductivities. For every 100th *I-V* cycle, we washed the devices. First 400 *I-V* cycles were measured with an applied voltage ±100 mV, and further cycles were in ±200 mV range. Inset shows the schematic of the respective Å-scale channels.

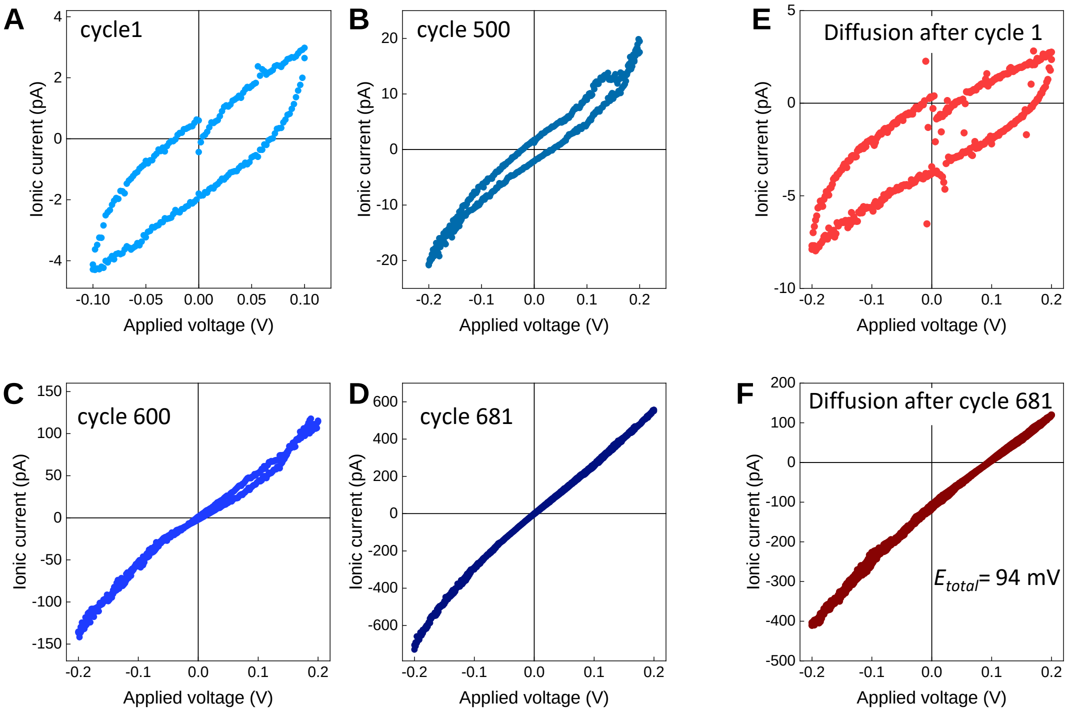

Fig. S6: I-V cycles and drift–diffusion measurements. (A to D) *I-V* characteristics of same device #9 in Fig.2 (in the manuscript), at several opening stages. **(E and F)** Drift – diffusion *I-V* characteristics of various states during opening, corresponding states indicated inside the graph. Device dimension: h = ~0.68 nm, w = ~110 nm, L = ~5 µm, n = 190.

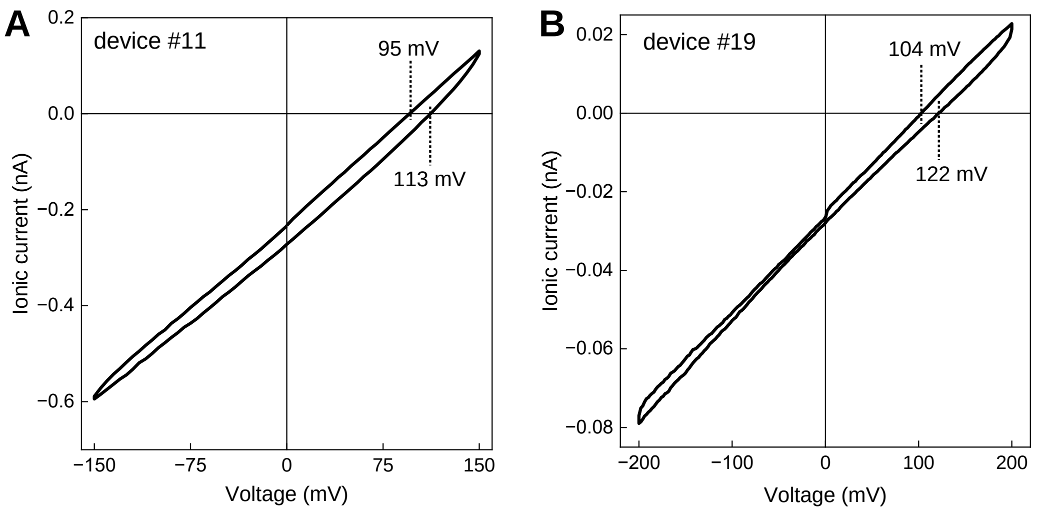

Fig. S7: Drift-diffusion curves for (A) device #11 and (B) device #19, respectively. The conductance data indicate that neither device opened fully. Additionally, the diffusion curves displayed hysteretic behaviour. Average values of E_{total} are presented in Fig. 3D of the main text.

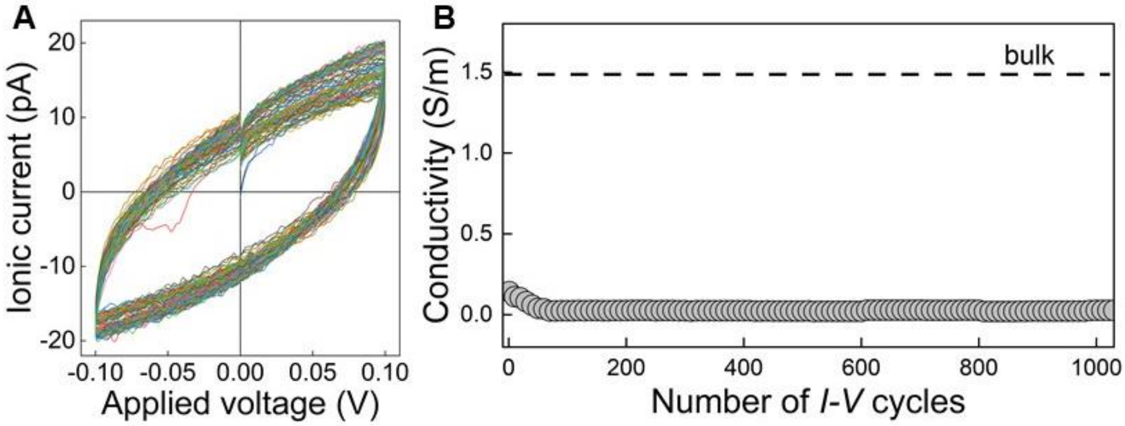

Fig. S8: Device opening for ±100 mV range *I-V* cycling. (A) *I-V* characteristic for 0.1 M KCl solution in the range of ±100 mV for 1000 cycles. **(B)** Conductivity was extracted for every 10th cycle out of 1000 cycles, with no change in conductivity observed over 1000 cycles. Washing was performed for every 100 cycles and replaced with fresh 0.1 M KCl solution. Dashed line represents the bulk[5] conductivity of 0.1M KCl. The Å-scale channels device used in this experiment containing bilayer graphene as a spacer (h ~ 6.8 Å), with top and bottom walls of hBN (n ~ 380 channels in parallel; w ~ 100 nm and L ~ 4.8 µm, device #32).

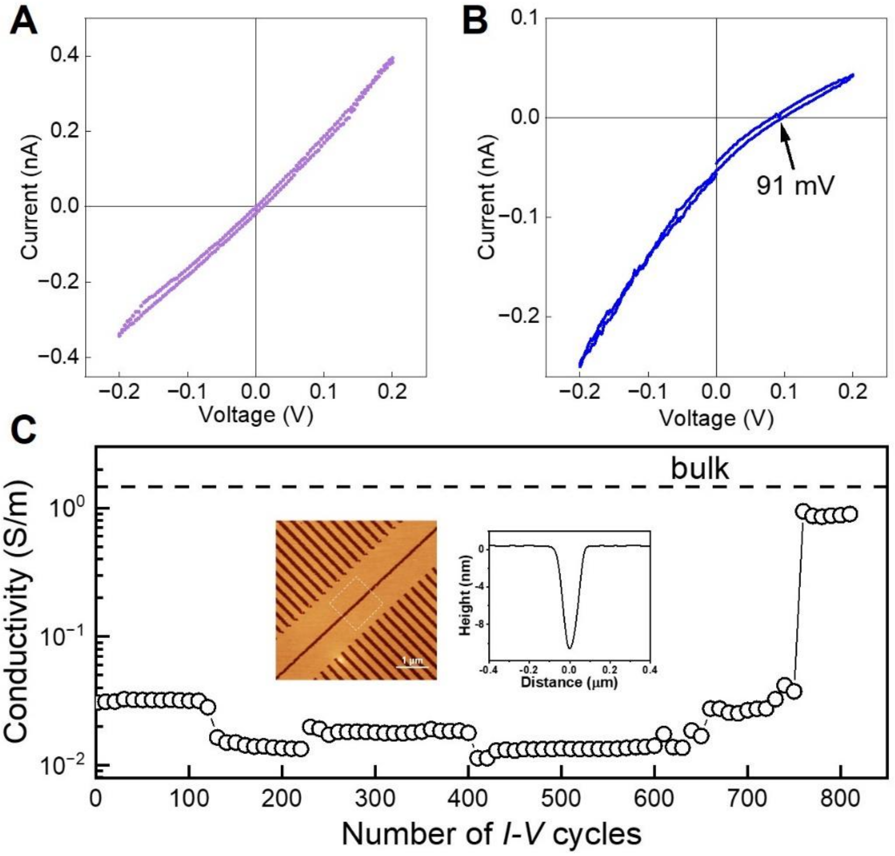

Fig. S9: Single–channel device. Figures illustrating (**A**) the *I-V* curve (0.1M KCl) and (**B**) drift-diffusion curve (C_H = 100 mM KCl and C_L = 10 mM KCl) for a single–channel device in the open state. (**C**) Comparison of the conductivity of the device in relation to the progression of *I-V* cycles. Device dimensions: $h \sim 12.4$ nm, $w \sim 80$ nm, $L \sim 500$ nm, $n \sim 1$, device #34. The first inset shows the AFM topography of a single-channel device, with the height profile displayed in the right-side inset figure corresponding to the white square box area.

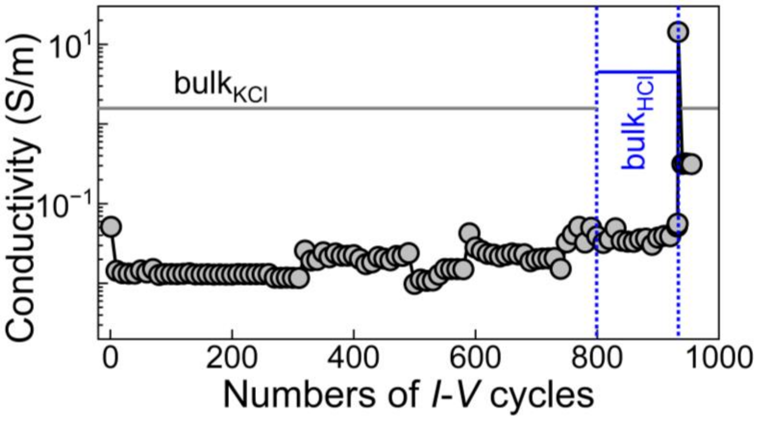

Figure S10. Conductivity *vs* number of *I-V* cycles for a device using 0.1 M KCl for 800 *I-V* (±200 mV range) cycles followed by 0.1 M HCl for 140 cycles (801 to 940 *I-V* cycle number, ±1 V range) and finally with 0.1 M KCl (941 to 955 *I-V* cycles, ±200 mV range). The grey and blue solid lines represent the bulk conductivity of KCl and HCl solutions, respectively at 0.1M concentrations. Device dimensions: h ~ 6.8 Å, w ~ 110 nm, L ~ 4.5μm, n ~ 198, device #33.

References

[1] a) B. Radha, A. Esfandiar, F. C. Wang, A. P. Rooney, K. Gopinadhan, A. Keerthi, A. Mishchenko, A. Janardanan, P. Blake, L. Fumagalli, M. Lozada-Hidalgo, S. Garaj, S. J. Haigh, I. V. Grigorieva, H. A. Wu and A. K. Geim, *Nature* **2016**, *538*, 222-225; b) S. Goutham, A. Keerthi, A. Ismail, A. Bhardwaj, H. Jalali, Y. You, Y. Li, N. Hassani, H. Peng, M. V. S. Martins, F. Wang, M. Neek-Amal and B. Radha, *Nature Nanotechnology* **2023**; c) A. Keerthi, S. Goutham, Y. You, P. Iamprasertkun, R. A. W. Dryfe, A. K. Geim and B. Radha, *Nature Communications* **2021**, *12*, 3092; d) A. Keerthi, A. K. Geim, A. Janardanan, A. P. Rooney, A. Esfandiar, S. Hu, S. A. Dar, I. V. Grigorieva, S. J. Haigh, F. C. Wang and B. Radha, *Nature* **2018**, *558*, 420-424; e) A. Bhardwaj, M. V. Surmani Martins, Y. You, R. Sajja, M. Rimmer, S. Goutham, R. Qi, S. Abbas Dar, B. Radha and A. Keerthi, *Nat Protoc* **2024**, *19*, 240-280.

[2] R. Sajja, Y. You, R. Qi, S. Goutham, A. Bhardwaj, A. Rakowski, S. Haigh, A. Keerthi and B. Radha, *Nanoscale* **2021**, *13*, 9553-9560.

[3] W. Steckelmacher, *Vacuum* **1966**, *16*, 561-584.

[4] J. Loessberg-Zahl, D. S. de Bruijn, W. T. E. van den Beld, E. Dollekamp, E. Grady, A. Keerthi, J. Bomer, B. Radha, H. J. W. Zandvliet, A. A. Bol, A. van den Berg and J. C. T. Eijkel, *The Journal of Physical Chemistry C* **2020**, *124*, 430-435.

[5] W. M. Haynes, *CRC Handbook of Chemistry and Physics*, CRC Press, **2003**, p.
